# A novel familial 9q31.2q32 microdeletion: Muscle cramping, somnolence, fatigue, sensorineural hearing loss, pubertal delay, and short stature

**DOI:** 10.1002/ccr3.1970

**Published:** 2019-01-07

**Authors:** Anand K. Ramineni, Trent Burgess, Penny Cruickshanks, David Coman

**Affiliations:** ^1^ Department of Paediatrics The Wesley Hospital Brisbane Queensland Australia; ^2^ Discipline of Paediatrics UnitingCare Clinical School Brisbane Queensland Australia; ^3^ Department of Neurosciences Lady Cilento Children's Hospital Brisbane Queensland Australia; ^4^ School of Medicine The University of Queensland Brisbane Queensland Australia; ^5^ Victorian Clinical Genetics Services Parkville Victoria Australia; ^6^ Murdoch Children's Research Institute Parkville Victoria Australia; ^7^ Department of Paediatrics University of Melbourne Parkville Victoria Australia; ^8^ Department of Paediatrics Sunshine Coast University Hospital Sunshine Coast Queensland Australia; ^9^ School of Medicine Griffith University Gold Coast Queensland Australia

**Keywords:** 9q microdeletion, delayed puberty, fatigue, FRRS1L, KLF4, sensorineural hearing loss, TXN, UCGC, ZNF48

## Abstract

We report a novel 9q31.2q32 (chr9: 109195179‐113974353, hg 18) microdeletion characterized by fatigue, muscle cramps, short stature, delayed puberty, sensorineural hearing loss, and mild developmental delay. Overlapping microdeletions reported in this region also demonstrate facial dysmorphism, skeletal anomalies, cleft palate, and cardiac valvular abnormalities. In comparing these cases, we suggest critical region of chr9: 109711873‐113407621 (hg 18).

## 1 INTRODUCTION

Interstitial microdeletions affecting the proximal 9q region are rare occurrences. However, the increasing use of chromosome microarray (CMA) testing has meant that precise breakpoints can be identified for patients affected by submicroscopic chromosomal rearrangements. This allows for closer delineation of critical regions and identification of candidate genes that may be central to the phenotype seen in deletion and duplication syndromes.^1^

Herein, we present a novel 9q31.2q32 microdeletion syndrome (chr9: 109195179‐113974353, hg 18) spanning several generations. This is the first report of this nature in the current literature and helps define a syndrome for proximal 9q deletions of fatigue, muscle cramps, short stature, delayed puberty, sensorineural hearing loss (SNHL), and mild developmental delay. The SNHL was bilateral, moderate to severe with 35‐113 dB loss across all frequencies. CMA techniques have been used in these patients to identify precise chromosomal breakpoints. These breakpoints were compared to other published cases analyzed with CMA.

## 2 CLINICAL REPORT

### 2.1 Index case

**Figure 1:**
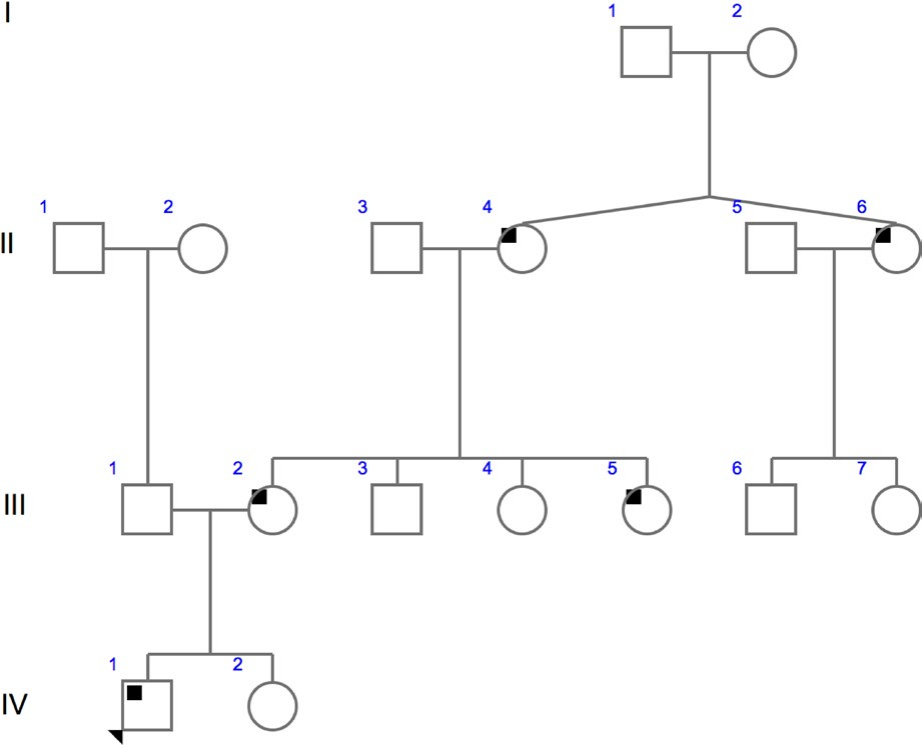
Family Genogram. Index case with arrow

The proband, IV1 in Figure 1, is the second child of non‐consanguineous parents and had an uncomplicated antenatal period being delivered at term. The patient presented for clinical genetic assessment at 11 years of age and is currently 19 years of age. He presented, at 11 years of age, with symptoms of muscle pain and cramps, beginning in the toes and progressing to the feet and calves. He experienced significant fatigue on most days with significant muscle weakness after light activity. He also has post‐lingual bilateral, moderate to severe SNHL of 35‐60 dB loss across all frequencies.

Neurological examination was unremarkable with normal gait, no proximal weakness, normal peripheral tone, power, reflexes, and sensation. Cranial nerve examination was also normal with no ptosis observed on sustained upward gaze. There was no clear evidence of fatigability consistent with a myasthenia syndrome, and subsequent nerve conduction studies were also normal. He was not dysmorphic, had a normal cardiac examination, and no clinical features to suggest a skeletal dysplasia. At 11 years of age, his weight at this stage was 29 kg (3rd percentile) and height 139.4 cm (1st percentile). His annual growth velocity at the time was 4.2 cm/y (<25th percentile). There were no signs of puberty. X‐rays were undertaken demonstrating a bone age of 11 years (10th percentile). A formal skeletal survey was normal with no radiological signs of a skeletal dysplasia. A growth hormone (GH) stimulation test was normal. Hormone testing revealed normal FSH and LH levels (both 1.8 mIU/mL) and a low testosterone level (1.2 mIU/mL). A testicular ultrasound measured his testes at 2.6 and 3.3 cm. He commenced pubertal induction with testosterone injections, which induced a 15 cm growth spurt to a height of 168 cm at 19 years of age.

**Figure 2:**
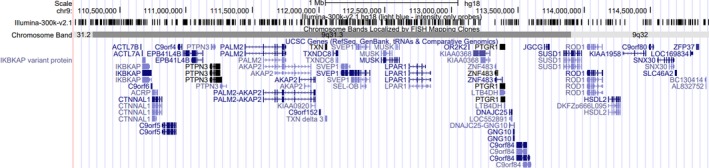
Graphical representation of the genes within the 9q31.2q32 microdeletion (chr9: 110,155,358‐114,938,931, hg18). Generated using the University of California Santa Cruz genome browser GRCh36/hg18 (https://genome.uscs.edu)

Normal results were obtained for creatine kinase, acylcarnitine profile, and mitochondrial DNA studies for common point mutations and deletion. CMA testing demonstrated a male profile with an approximate 4.8 Mb interstitial deletion on chromosome 9 from band 9q31.2 to 9q32 (chr9: 109195179‐113974353, hg 18). This microdeletion including gene content is depicted in Figure 2.

### 2.2 Other affected family members

The same 9q31.2q32 microdeletion was segregating amongst the index case's family members with clinical features as summarized in Table 1. Patient III5 is notable for significant fatigue symptoms with excessive daytime somnolence and muscle cramps. She has primary hypersomnia and had a favorable response to modafinil. She was also referred to a neurologist and underwent nerve conduction studies to assess for a potential myasthenia syndrome, like those undertaken by Patient IV1. The results of these were normal. SNHL in affected individuals was post‐lingual, bilateral, moderate to severe SNHL of 45‐113 dB loss across all frequencies.

**Table 1:** Phenotype comparison of patient with familial 9q31.2q32 microdeletion (chr9: 110155358‐114938931, hg18)

	Patient IV1	Patient IV2	Patient II4	Patient II6	Patient III2	Patient III5
Current age	19	22	73	73	47	33
Gender	Male	Female	Female	Female	Female	Female
Fatigue	Yes	No	No	No	No	Severe
Muscle cramps	Yes	No	Yes	No	No	No
Short stature, cm	168	165	153	147	156	152
Delayed puberty (age onset)	15	16	13	15.5	16	14.5
Menopause (age onset)	N/A	N/A	50	50	N/A	N/A
Sensorineural hearing loss	Right 60 dB Left 35 dB	No	No	No	+ tinnitus Right 45 dB Left 50 dB	Right 47 dB Left 113 dB
Delayed motor development	Yes	No	Yes Walked at 20 mo	Yes Walked at 20 mo	No	Yes Walked at 22 mo
Learning difficulties	Dyslexia	No	Retaining information	No	Retaining information	Retaining information + dyscalculia
Anxiety	Yes	Yes	Yes	No	Yes	Yes
Other	No	No	Pyloric stenosis	Pyloric stenosis + strabismus	No	Primary hypersomnia + VSD

N/A, not applicable; VSD, ventricular septal defect.

**Figure 3:**
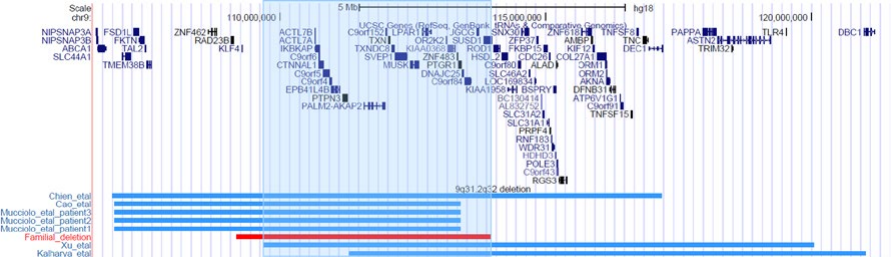
Graphical representation of the 9q31.2q32 microdeletion in Patients 1‐6 (chr9:110,155,358‐114,938,931, hg18) compared to patients in the literature with overlapping deletions. All patient breakpoints were converted to build GRCh36/hg18 (https://genome.uscs.edu) for direct comparison

Patient numbers align with the pedigree in Figure 3.

## 3 MATERIALS AND METHODS

DNA was extracted from peripheral blood samples, and CMA was performed using Illumina HumanCytoSNP‐12 version 2.1.^1^ All procedures for fragmentation, labeling, and hybridization were performed according to the manufacturer's protocol. Raw data were processed using Karyostudio (Illumina, San Diego, CA, USA), and probe intensity measurements were normalized to a reference set of 100 clinical samples. The significance of each copy number detected was determined by comparison with public databases of copy number variants (ie, Children's Hospital of Philadelphia [CHOP], International Standards for Cytogenomic Arrays [ISCA], and the Database of Genomic Variants [DGV] and an internal set of clinical samples). Analysis was performed using UCSC Genome Browser March 2006 hg18 assembly. The resolution of the CMA for copy number detection is set at 0.20 Mb.

## 4 DISCUSSION

Interstitial deletions affecting the long arm of chromosome 9 are rare occurrences. Approximately 20 cases have been published in the literature describing deletions overlapping the 9q31.2q32 region identified in our cohort. Most of these were detected using conventional chromosome banding analysis, and to date, no clear syndrome has been identified^2^, ^3^, ^4^, ^5^, ^6^, ^7^, ^8^). Seven cases have currently been reported using CMA techniques with precise breakpoints that overlap the 9q31.2q32 region.^9^, ^10^, ^11^, ^12^, ^13^ These are represented graphically in Figure 3 and aligned against the deletion seen in our cohort, with a summary of the clinical reports available in Table 2. A critical overlapping area extends from chr9: 109711873‐113407621 (hg18). Overlapping reported phenotypic features include short stature, SNHL, pubertal delay, and developmental delay. Many of the cases reported using conventional banding techniques also have markedly similar clinical findings, with most patients reported to have hearing loss, short stature, developmental delay, and poor growth^2^, ^3^, ^4^, ^5^, ^6^, ^7^, ^8^). However, direct comparison is problematic due to the subjectivity associated with assigning chromosomal bands during conventional chromosome analysis.

**Table 2:** Phenotype comparison of reported cases with interstitial deletions overlapping 9q31.2q32

	Current cases	Kulharya et al	Mucciolo et al Case 1	Mucciolo et al Case 2	Mucciolo et al Case 3	Chien et al	Xu et al	Cao et al
Interstitial deletion	9q31.2q32	9q31.1q33.1	9q31.1q31.3	9q31.1q31.3	9q31.1q31.3	9q31.1q31.3	9q31.2q33.1	9q31.1q32
Chromosome 9 breakpoints	110155358‐114938931	111303224‐121018591	107858730‐114367800	107945742‐114439602	107945742‐114439602	106859697‐117190101	110672052‐120997503	105104179‐117250435
Other genetic anomalies	−	−	−	−	−	Ins (18;9)(q12;q33.1q31.1)	ANKRD11 mutation	−
Digital anomalies	−	+	+	NR	NR	+	+	NR
Cervicothoracic gibbus	−	NR	+	+	+	NR	NR	NR
Poor growth	+	+	−	−	−	+	+	+
Short stature	+	+	+	+	+	NR	+	+
Delayed puberty	+	NR	NR	NR	NR	NR	+	NR
Sensorineural hearing loss	+	NR	−	NR	+	+	+	+
Seizures	−	+	−	NR	NR	NR	−	NR
Developmental delay	+	+	+	−	+	+	+	+
Cardiac anomalies	+	NR	+	+	−	+	+	+
Strabismus	+	NR	NR	NR	NR	+	−	NR
Dysmorphic facial features	−	+	+	+	+	+	+	+
Cleft lip/palate	−	NR	−	−	NR	+	NR	NR

+, present; −, absent; NR, not recorded.

Kulharya et al^11^ reported a patient with a 9q31.1q33.1 deletion (chr9: 111303224‐121018591, hg18) with a phenotype of developmental delay, poor growth, short stature, and dysmorphic facial features. Mucciolo et al^12^ described three patients with deletions affecting different breakpoints involving this region. The first had an approximate 6.5 Mb deletion involving chromosome region 9q31.1q31.3 (chr9: 106898551‐113407621, converted to hg18 from hg19) and showed delayed motor milestones, dysmorphic facial features, short stature, cervicothoracic gibbus, and aortic insufficiency. The remaining two patients were monozygotic twins who shared an approximate 6.5 Mb deletion of almost the same chromosome region (chr9: 106898551‐113407621, converted to hg18 from hg19). Twin 1 had dysmorphic facial features, learning difficulties, dilated cardiomyopathy, short stature, hepatosteatosis, and cervicothoracic gibbus. Twin 2 had similar dysmorphic facial features, learning difficulties, short stature, cervicothoracic gibbus, and sensorineural hearing loss. Chien et al^10^ described a patient with a 10.3 Mb deletion involving chromosome region 9q31.1q33.1 (chr9: 106859697‐117190101, hg18) due to a familial insertion ins(18;9)(q12.2;q33.1q31.1, hg18). Clinical findings included cleft lip and palate, delayed motor milestones, intellectual impairment, poor growth, dysmorphic facial features, and sensorineural hearing loss. Ying et al^13^ reported a case with a 10.3 Mb deletion in chromosome region 9q31.2q33.1 (chr9: 109711873‐120037324, converted to hg18 from hg19) as well as a heterozygous missense mutation in *ANKRD11* (OMIM 611192) on chromosome 16. This patient had the features of KBG syndrome (OMIM 148050). Cao et al^9^ described a patient with a 12.1 Mb deletion involving chromosome region 9q31.1q32 (chr9: 106898551, converted to hg18 from hg19) with sensorineural hearing loss, poor growth, delayed motor milestones, and dysmorphic facial features. Comparison was made between this patient's phenotype and the clinical features of Cornelia de Lange syndrome (CdLS, OMIM 122470). The dysmorphic features in the aforementioned cases have included prominent forehead, bulbous nose, epicanthic folds, small mandible, short philtrum, thick hair, and arched eyebrows. Our patient cohort share similarities of short stature, delayed puberty, SNHL, and mild developmental delay; however, they are not dysmorphic and do not have skeletal anomalies. Figure 2 compares the reported breakpoints, and we suggest a critical region of chr9: 109711873‐113407621 (hg 18).

Our cohort of novel 9q31.2q32 deletions reveals several candidate genes in which haploinsufficiency may correlate with the phenotypic findings. The *KLF4* gene (Kruppel‐like factor 4, OMIM 602253) is located at 9q31.2 (chr9: 109286956‐109291576, hg18). Kruppel‐like factor 4 is a transcription factor mainly expressed in the epithelium of the intestine and skin. It has diverse functions in tissue homeostasis and can act as a transcriptional activator/repressor, tumor suppressor, and oncogene.^14^ It also has important functions in cell differentiation and has been shown to help transform mouse fibroblasts into an embryonic‐like state of pluripotency.^15^ Djalilian et al^16^ have demonstrated that mice lacking *KLF4* have severely underdeveloped epidermal barriers and abnormal expression of the gap junction protein *Cx26* (connexin 26, OMIM 121011). Mutations in *Cx26* have been linked to sensorineural hearing loss in humans, and altered expression has been shown to adversely affect development of inner hair cells in mouse cochlea.^17^ With sensorineural hearing loss being a prominent finding in 9q31.2q32 deletions, haploinsufficiency of *KLF4* and subsequent altered expression of *Cx26* may be of pathogenic significance.

The *TXN* gene (thioredoxin, OMIM 187700) located at 9q31.3 (chr9: 112046131‐112058599, hg18), thioredoxin system and associated isoenzymes are oxidoreductases that scavenge free radicals and play a key role in regulation of transcription and cell growth.^18^ Thioredoxin is kept in a reduced state by thioredoxin reductase (*TXNRD1*, OMIM 601112) and its related isoenzymes. Dammeyer et al^19^ found that *TXNRD1* was strongly expressed in rat cochlea and that exposure to cisplatin and oxaliplatin significantly reduced *TXNRD1* activity. Tadros et al^20^ demonstrated that *TXNRD1* expression was downregulated in mice cochlea with age‐related hearing loss. These results suggest that the thioredoxin system may play a key role in hearing loss in 9q31.2q32 deletions.

The *UGCG* gene (UDP‐glucose ceramide glucosyltransferase, OMIM 602874) located at 9q31.3 (chr9: 113699027‐113735254, hg18) and its gene product catalyses the first step of glycosphingolipid synthesis forming glucosylceramide. This serves as the core component of hundreds of glycosphingolipids, which are important cell membrane components.^21^ Rabionet et al^22^ demonstrated that *UGCG* was important in the maturation of sperm‐specific glycosphingolipids. Deletion of *UGCG* in mouse germ cells caused age‐dependent reduction in testicular mass, tubular atrophy, and arrested spermatogenesis. This is of relevance as delayed puberty is one of the common phenotypic features in many male cases with 9q31.2q32 deletions. Our index case was found to have small testes, low testosterone, and a requirement for pubertal induction with testosterone injections.

*ZNF483* is located at 9q31.3 (chr9: 113327268‐113346533, hg18) and while scant information exists as to its function beyond potential transcriptional regulation,^23^ numerous groups have identified *ZNF483* to be associated with age at menarche in women.^24^, ^25^, ^26^, ^27^, ^28^ A key clinical feature of the female patients in our cohort is that of delayed puberty, potentially reflecting the importance of *ZNF483* in the normal onset of puberty.

The *FRRS1L* gene (ferric chelate reductase 1 like, OMIM 604574) is located at 9q31.3 (chr9: 110939402‐110969268, hg18) and encodes a component of α‐amino‐3‐hydroxy‐5‐methyl‐4‐isoxazolepropionic acid (AMPA) receptors, which are mediators of excitatory glutamatergic neurotransmission. Mutations in *FRRS1L* have been shown to impair AMPA‐mediated neurotransmission and lead to an early infantile epileptic‐dyskinetic encephalopathy.^29^ *FRRS1L* may be of relevance when considering the propensity of Patients 1‐6 to fatigue symptoms, and specifically that Patient III5 symptoms were responsive to modafinil therapy. Modafinil is used in the treatment of excessive somnolence in conditions such as narcolepsy and obstructive sleep apnea. It acts by inhibiting central dopamine and noradrenaline uptake, subsequently increasing extracellular concentrations of dopamine, noradrenaline, serotonin, and glutamate.^30^ Modafinil also increases receptor complex levels of several AMPA subtypes in mice.^31^ Given that modafinil was effective in a patient with *FRRS1L* haploinsufficiency, this may suggest a link between *FRRS1L*, AMPA, and fatigue symptoms in 9q31.2q32 deletions.

With relation to fatigue symptoms, it is also important to consider the *MUSK* gene (muscle‐specific receptor tyrosine kinase, OMIM 601296) which is located at 9q31.3 (chr9: 112470908‐112603099, hg18). *MUSK* encodes kinase proteins that are essential for normal signaling across the neuromuscular junction. Autosomal recessive mutations in *MUSK* have been associated with congenital myasthenic syndromes^32^; thus, haploinsufficiency would not be expected to generate clinical symptomatology. Nerve conduction studies and electromyography performed on Patient's IV1 and III5 and 6 were normal suggesting that their fatigue symptoms do not relate to *MUSK* haploinsufficiency.

In conclusion, our cohort represents a novel contiguous proximal 9q microdeletion which further defines a syndrome for deletions affecting this area. It also narrows a critical region when compared to similar reported cases and provides support for haploinsufficiency of several candidate genes, especially those linked with SNHL, fatigue, and timing of pubertal onset.

## CONFLICT OF INTEREST

None declared.

## AUTHOR CONTRIBUTION

DC and PC: are primary caregivers for the family. TB: performed all the cytogenetic work. AR, TB, PC, and DC: all contributed to writing the manuscript. DC: provided final review and editing.
